# Reciprocity in breast cancer progression

**DOI:** 10.18632/oncotarget.2853

**Published:** 2014-12-01

**Authors:** Christian Gespach

**Affiliations:** INSERM U938, Molecular and Clinical Oncology, Hôpital Saint-Antoine, Paris, France

Progression of solid tumors is characterized by an evolutive growing cellular mass containing variable proportions of a complex network of tumor stroma cell populations (TSC, about 30-70% in colon cancer) surrounding cancer cell (CC) foci. This cellular intricacy determines mechanical, molecular and functional interactions involved in multiple mechanisms driving reciprocal aspects of paracrine and justacrine crosstalks between TSC and adjacent CC. There is increasing evidence that tumor stroma is a multicellular organ containing vascular structures and a large variety of cellular populations, including resident carcinoma-associated fibroblasts (CAF), myofibroblasts (MF), leucocytes and macrophages, bone marrow-derived mesenchymal stem cells, and in breast cancer, adipose progenitor cells. This cellular heterogeneity sustains tumor growth and angiogenesis, deregulated cancer cell proliferation, survival, chemoresistance, epithelial-to-mesenchymal transitions (EMT), invasion and tumor metastasis.

In primary tumors and their metastases, these intercommunications between cancer cells and their surrounding TSC are organized in part by the convergent secretion of various ECM components building the architectural formation of ECM interfaces between these two critical cellular populations. ECM remodeling during cancer progression is regulated by the concomitant secretion of CC and TSC soluble factors and cytokines as well as subcellular exosomes involved in genetic tranfer between cancer cells and their stromal microenvironment (reviewed in ref. [1]). Plasticity of the ECM and secretome molecules and structures at the CC/TSC interface reciprocally modify CC and TSC gene expression, differentiation and other aspects of carcinogenesis, including cancer cell adhesion and spreading.

Several studies pointed the critical roles played by the ECM, CAF and MF in tumor progression. Decorin is a member of the Small Leucine-rich Repeat Proteoglycan (SLRP) family expressed and secreted in the interstitial ECM in breast stroma (reviewed in ref. [2]). In ECMs, the multifunctional protein decorin is associated with fibrillar collagens type I, II, III and VI and contributes in matrix organisation and architecture. Decorin is also localized at the cell plasma membrane where it interacts with cell surface receptors or ligands. Of note, decorin is currently considered as an anti-cancer agent by suppressing a series of signaling pathways. Decorin protein core causes a long-term blockade or endocytosis of tyrosine kinase receptors (HER1, HER2, IGFR, MET), chemokine G-protein receptors CXCR4, LDL receptor-related protein (LRP-1), and α2β1 integrins [2]. These decorin-dependent signal transduction systems are implicated in mitogenic and oncogenic functions connected with cancer cell adhesion, invasion, tumor angiogenesis and metastasis. Consistently, decorin suppression in decorin knock-out mice is permissive for tumor development [2].

In this context, decorin was shown to sequester latent form of TGFβ1 (L-TGFβ1) in the ECM and to interact with the active TGFβ1 ligand, thus preventing its binding to TGFβ1 receptors [2]. Most importantly, excessive ECM remodeling induced by mechanical stress during fibrosis and cancer leads to the release of the bioactive form of the cytokine [3]. TGFβ1 signals are transduced by TGFβRII-dependent TGFβRI signaling to canonical cytoplasmic and nuclear Smad proteins involved in transcriptional responses. A vast array of cytoplasmic and nuclear TGFβ pathways using non-Smad elements is also described, such as Rho-GTPases, stress-activated protein kinases JNK/p38, MAPK, and the c-JUN/c-FOS components of AP1-dependent transcription (reviewed in ref. [4]). TGFβ1 plays opposing roles in tumor progression depending upon the stages of the disease, leading to protection during normal development versus promotion/progression at the premalignant/carcinoma transitions. The oncogenic activities of TGFβ1 are covered by acquisition of deleterious cellular dysfunctions associated with carcinogenesis as illustrated by the uncontrolled cell division, resistance to apoptosis, EMT and CAF/MF transitions conversion, invasive and metastatic cascades.

In the background of ductal carcinoma in situ (DCIS) of the breast, Van Bockstal et al reveal that the function of decorin is involved in breast cancer cell spreading and that both TGFβ1 and bFGF down-regulated the ECM protein decorin in CAF-associated breast tumors [5]. In turn, breast cancer cells showed a significant enhanced spreading when plated on TGFβ1-treated, decorin-depleted, CAF-associated ECM. Thus, TGFβ1 secreted in tumor stroma of DCIS is described as a critical permissive factor to initiate proinvasive pathways through ECM decorin deficiency in CAF. In breast cancer, reduced stromal decorin correlates with the myxoid stromal architecture, both are associated with increased recurrence risk in DCIS and propensity to progress to invasive ductal carcinoma (IDC). The authors suggested that this TGFβ1-induced decorin repression in CAF is involved in the mechanisms driving transition of DCIS into IDC of the breast. In support with this conclusion, TGFβ1 was previously reported to reduce decorin mRNA and protein levels through unidentified mechanisms in human dermal fibroblasts in culture [6,7].

**Figure 1:**
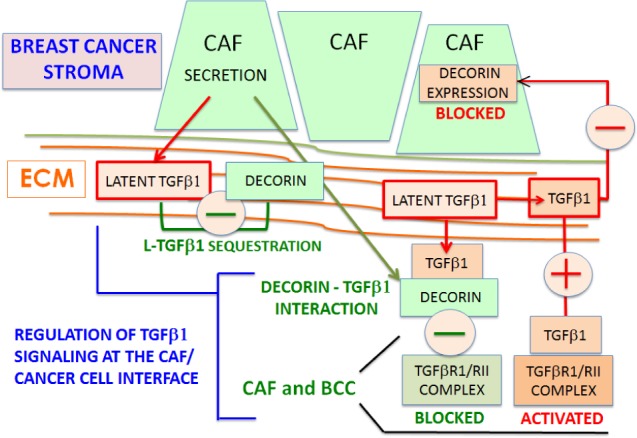
Reciprocal Decorin-TGFβ1 interplay in breast cancer progression

These findings collected in breast cancer-associated fibroblasts [5] are of paramount importance in order to elucidate further the pathophysiological mechanisms sustaining the functional reciprocity between TGFβ1 and decorin inhibitory actions (Figure 1). Identification of the signaling pathways and mechanisms regulating TGFβ1-induced decorin down-regulation in CAF/MF *in vitro* and *in vivo* will provide a rationale for new therapeutic options aimed to neutralize the oncogenic role of TGFβ1 as a repressor of the decorin tumor suppressive functions. As reported by Van Bockstal *et* al [5] this assumption is also valid for the inhibitory effects of bFGF on decorin expression in breast cancer-associated fibroblasts.
